# Screening Mammography & Breast Cancer Mortality: Meta-Analysis of Quasi-Experimental Studies

**DOI:** 10.1371/journal.pone.0098105

**Published:** 2014-06-02

**Authors:** Veronica L. Irvin, Robert M. Kaplan

**Affiliations:** Department of Rehabilitation Medicine, Clinical Research Center, National Institutes of Health, Bethesda, Maryland, United States of America; CUNY, United States of America

## Abstract

**Background:**

The magnitude of the benefit associated with screening has been debated. We present a meta-analysis of quasi-experimental studies on the effects of mammography screening.

**Methods:**

We searched MEDLINE/PubMed and Embase for articles published through January 31, 2013. Studies were included if they reported: 1) a population-wide breast cancer screening program using mammography with 5+ years of data post-implementation; 2) a comparison group with equal access to therapies; and 3) breast cancer mortality. Studies excluded were: RCTs, case-control, or simulation studies. We defined quasi-experimental as studies that compared either geographical, historical or birth cohorts with a screening program to an equivalent cohort without a screening program. Meta-analyses were conducted in Stata using the metan command, random effects. Meta-analyses were conducted separately for ages screened: under 50, 50 to 69 and over 70 and weighted by population and person-years.

**Results:**

Among 4,903 published papers that were retrieved, 19 studies matched eligibility criteria. Birth cohort studies reported a significant benefit for women screened <age 50, but not for women screened ages 50–69. Significant reductions in breast cancer mortality were observed in historical comparisons. For geographical comparisons, there was a significant 20% reduction in mortality for women <age 50 and a significant 21–22% reduction for women ages 50–69. Studies that tested the interaction of geographical and historical comparisons produced a pooled, significant 13–17% reduction in incident breast cancer mortality for women ages 50–69, but the effects in most individual studies were non-significant. All studies of women ages 70+ were non-significant.

**Conclusions:**

Mammography screening may have modest effects on cancer mortality between the ages of 50 and 69 and non-significant effects for women older than age 70. Results are consistent with meta-analyses of RCTs. Effects on total mortality could not be assessed because of the limited number of studies.

## Introduction

Screening for breast cancer using mammography remains one of the most controversial issues in contemporary medicine and health care. Mammography screening may be the best approach to reduce the burden of premature death associated with breast cancer [1]–[4]. However, cancer screening can cause harm as well as improvement and women may not often have a balanced presentation of risks and benefits [5], [6].

The evidence base for assessing the risks and benefits of mammography gives greatest weight to randomized clinical trials (RCTs). RCTs on mammography are expensive and difficult to conduct. As a result, a limited number of RCTs have been completed and most of the debate focuses on 8 large trials [7]–[11]. Multiple systematic reviews have been conducted and their conclusions shift as a function of which RCTs are included in the analysis. These systematic reviews and expert, independent reviews have found significant 15–21% reductions in breast cancer mortality for women screened between ages 50–69 [7]–[13] The U.S. Preventive Services Task Force (USPSTF) reported significant reductions in breast cancer mortality in women screened below age 50 in addition to those screened above age 50. Sufficient data was lacking to draw conclusions about screening women age ≥70 [8]. These meta-analyses did not show a benefit of screening for any age group or follow-up length if all-cause mortality was the outcome measure [7], [8], [14]. Twenty-year follow-up data from the Swedish Two-County Trial showed a 13% reduction in deaths from all causes among breast cancer cases [15]. An independent review panel in the U.K. estimated that a 20% relative risk reduction in breast cancer deaths for ages 55–79 would yield a 1.2% reduction in all-cause deaths. However, the trials do not have enough number of women or years of follow-up to reliably estimate these small relative risk values [9].

The debate over the evidence has become stagnant because no new RCTs have recently been completed nor are there new trials in progress. Most of the new evidence comes from non-experimental trials or quasi experiments. Although many believe that the RCT is the only way to evaluate the benefits of treatment, RCTs also have well known methodological problems for evaluating population-based health interventions. RCTs are expensive and often based on participants who are not-representative of the population that might result in poor external validity [16], [17] and can lack internal validity through failure of proper randomization, loss to follow-up and misclassification of end points [9]. Further, treatments evaluated in RCTs may not be representative of those delivered in clinical practice.

In contrast to RCTs, natural experiments (or quasi-experiments) often evaluate policy and are able to avoid selection biases. They are typically representative of the care people receive in the community and of the subject populations to which the results will be generalized. Participants are often assigned to treatment for quasi-random reasons. Governments may only have sufficient resources to introduce mammography in one community. A comparable neighboring community without a screening program might serve as a reasonable control.

Previous systematic reviews of observational or quasi-experimental studies included case-controls or did not prepare a meta-analysis of study effects [18]–[21]. Two recent meta-analyses of observational studies for mammography screening of women 50–69 were published, but they either combined effects for studies of varying designs or did not pool the effects for all studies in the meta-analyses [22], [23]. Neither study reported on studies of women screened younger than 50 or older than 69 [22], [23]. This paper presents meta-analyses of different types of quasi-experimental studies: comparing birth cohorts, creating historical control groups, or taking advantage of geographic natural experiments by age screened. This is the first review of quasi-experimental studies to include women screened under age 50 as well as over age 70.

## Methods

### Data sources and searches

**Figure 1:**
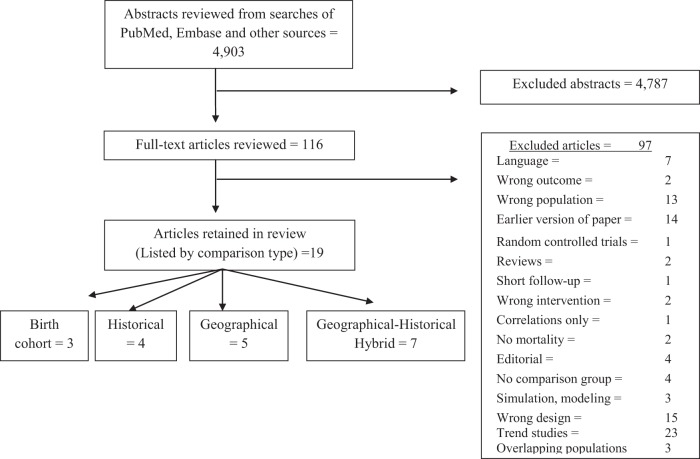
PRISMA Flow diagram. Number of articles excluded and reviewed for inclusion in meta-analysis.

Using a broad search strategy, electronic searches were conducted of MEDLINE/PubMed and Embase for articles published up through January 31, 2013 (no start date). The detailed search strategy and the number of studies produced with each strategy are provided in Table S1 and Checklist S1. Figure 1 shows the PRISMA flow diagram of the number of searches returned, excluded and reviewed. The final searches yielded 4,903 citations: 2,249 PubMed and 2,654 in Embase after removing duplicates. The number of articles identified exceeds that in previous meta-analyses. Secondary referencing was conducted by manually searching bibliographies from meta-analyses and other systematic reviews. Abstracts and titles were read and were eliminated if inclusion criteria were not clearly met. When unclear, articles were reviewed in full. Table S2 lists the number and reasons for abstracts that were excluded from full review.

### Study eligibility

Studies were included if they reported: 1) a population-wide breast cancer screening program (the population could be city, county, or nation) with at least 5 years of study data post-implementation; 2) a comparison group with equal access to breast cancer therapies; and 3) breast cancer mortality. Studies excluded were: RCTs, case-control, simulation studies or modeling studies; studies that compared trends but did not provide mortality numbers; studies that compared only clinical breast or self-breast exam; studies that compared self-selected participants to non-participants; and studies of high risk groups or only women diagnosed with breast cancer. Studies that compared observed deaths to expected deaths were excluded because the expected numbers of deaths were based off of modeling.

Studies from the same country were retained as long as there was no overlap between the population, region or time period studied. When a study reported multiple comparisons in the same paper, the comparison with the larger screening population was retained.

If multiple studies compared the same region, population and time period, typically the study with the longest follow-up period was retained. Below are the specifics of the overlapping studies that were removed. Hakama et al conducted birth cohort analyses of mammography screening in Finland with follow-up at 6 and 9 years [24], [25]. The six year follow-up [24] was reported because the purpose of the 9-year follow-up [25] was to demonstrate the effect of gradually screening women originally assigned to control population (Personal communication, Matti Hakama, 10/30/12). Three manuscripts compared similar but not exact Swedish municipalities. The SOSSEG, 2006 paper was retained because it reported the longest follow-up; Duffy et al., 2002 and Tabar et al., 2003 were not included because of their significant overlap with it [26]–[28]. Although Jorgensen et al., 2010 reported the longer follow-up, Olsen et al., 2005a was retained in the primary meta-analysis because it analyzed incidence-breast cancer mortality [29], [30]. Van Dijick et al., 1997 and Broeders et al., 2001, both reported on screening in Nijmegen, the Netherdlands, but Van Dijick was retained in the primary analyses because it analyzed incident breast cancer mortality and reported relative risks without adjustments [31], [32]. Similarly, Olsen et al., 2012 and Kalager et al., 2010 reported on similar geographical regions in Norway, but Olsen et al., 2012 was retained in the primary analyses because it had a longer follow-up period [33], [34].

### Data extraction

VI reviewed all abstracts; while RK confirmed all included abstracts and those that were undecided. Both authors reviewed all potential full articles. When clarification was needed, the corresponding author for that study was contacted. 14 study authors were contacted with questions and 9 responded. Two study authors were not contacted because all necessary data were available in their publication. Data analyses were conducted by VI and both investigators drafted the manuscript.

### Ethics Statement

This study was determined exempt from review by the National Institutes of Health, Office of Human Subjects Research.

### Statistical analysis

For each study retained in the meta-analysis, data extracted include (Table 1): number of breast cancer deaths, population size, number of years of follow-up, person-years, and unadjusted relative risks and confidence intervals that were published and those re-calculated by the study authors (i.e. not adjusted for attendance, lead time or inclusion bias). Incident-based breast cancer mortality (i.e. refined breast cancer mortality) excludes women diagnosed with breast cancer before the start of screening program in all study and comparison populations. Prevalent-based breast cancer mortality retains these women. Incident and prevalent breast cancer outcomes were reported separately.

**Table 1:** Screening details, relative risks and confidence intervals for included studies by study design.

Author	Country	Time period of accrual	Time period of screening	Time period of follow-up	Screening Interval	Attendance rates	Type of exam	Age group screened	Breast Cancer Mortality RR (95% CI)		
									Published	Re-calculated	
**Study design: Birth cohort comparisons**											
Anttila, 2002	Finland	1986–1997	1986–1997	1986–1997	2 years	82%	2-view	50–59	0.81 (0.62,1.05)		0.80 (0.57,1.13)
Hakama, 1995	Finland	1982–1983	1982–1989	1982–1991	2 years	86%	1-view	<50	0.11 (0.00,0.71)		0.11 (0.01,0.85)
Hakama, 1997	Finland	1987–1989	1987–1992	1987–1995	2 years	90%	2-view	50–59	0.76 (0.53,1.09)		0.76 (0.54,1.07)
**Study design: Historical comparisons**											
Ascunce,2007^a^	Spain	1997–2001	1991–2001	1997–2001	2 years	88%	NR	45–69	0.58 (0.44,0.75)		0.57 (0.44,0.47)
Duffy, 2010^b, h, j^	UK	1989–2004	1989–2004	1995–2004	3 years	70%	1 or 2-view	50–64	0.73 (0.72,0.74)		0.73 (0.72,0.74)
Otto, 2003^b, c, h, i^	Netherlands	1989–1997	1989–1999	1989–1999	2 years	78%	2-view	50–69	NR		0.97 (0.95,1.00)
SOSSEG, 2006^i^	Sweden	1980–2000	1980–2000	1980–2000	NR	63–93%	NR	40–69 or 50–69	0.73 (0.69,0.77)		0.72 (0.68,0.76)
**Study design: Geographical comparisons**											
Hellquist, 2011^i^	Sweden	1986–2005	1986–2005	1986–2005	1–2 years	80–90%	1 or 2-view	40–49	0.79 (0.72,0.86)		0.79 (0.72,0.86)
Jonsson, 2007^d^	Sweden	1990–1996	1990–2001	1997–2001	1–2 years	83–87%	1 or 2-view & ultrasound	50–69	0.80 (0.64,1.00)		0.80 (0.64,1.00)
Jonsson, 2007^d, i^	Sweden	1990–1996	1990–2001	1997–2001	1–2 years	83–87%	1 or 2 -view & ultrasound	70–74	0.97 (0.62,1.52)		0.97 (0.62,1.52)
No authors, 1999^c, g, k^	UK	1980–1983	1980–1995	1980–1995	2 years	60–72%	Varied	45–64	0.73 (0.63,0.84)		0.78 (0.69, 0.87)
Peer, 1995	Netherlands	1975–1976	1975–1990	1975–1990	2 years	87%	1-view	35–49	0.94 (0.68,1.29)		0.94 (0.68,1.30)
Van Dijck, 1997	Netherlands	1977–1978	1977–1990	1977–1990	2 years	46%	1-view	68+	0.80 (0.53,1.22)		0.80 (0.53,1.22)
**Study design: Geographical-historical hybrids**											
Jonsson, 2000	Sweden	1986–1996	1986–1996	1986–1996	1–2 years	NR	NR	40–49	0.91 (0.72,1.15)		0.91 (0.72,1.15)
Jonsson, 2001^i^	Sweden	1986–1994	1986–1994	1986–1997	NR	NR	NR	50–69	0.91 (0.74,1.10)		0.90 (0.74,1.10)
Jonsson, 2003^e, i^	Sweden	1974–1986	1974–1986	1977–1998	2–3 years	84%	1-view	40–64	0.93 (0.77,1.11)		0.93 (0.78,1.11)
Jonsson, 2003^ i^	Sweden	1986–1998	1986–1998	1986–1998	1–2 years	NR	NR	70–74	0.97 (0.73,1.28)		0.97 (0.74,1.28)
Olsen, 2005	Denmark	1991–2001	1991–2001	1991–2001	2 years	71%	NR	50–69	0.74 (0.63,0.89)		0.73 (0.62,0.87)
Olsen, 2013	Norway	1996–2002	1996–2002	1996–2008	2 years	NR	NR	50–69	0.93 (0.77,1.12)		0.90 (0.75,1.09)
Parvinen, 2006^f^	Finland	1987–1997	1987–1997	1987–2001	2 years	NR	NR	55–69	0.58 (0.41,0.83)		0.58 (0.41,0.82)

NR = Not reported.

A. Most studies reported mortality as incidence-based breast cancer mortality. These studies report both incidence and prevalence-based breast cancer mortality. Numbers in cells represent incidence-based mortality.

B. Studies report only prevalence-based breast cancer mortality. Numbers shown reflect prevalence-based mortality numbers and RR calculations.

C. For the Otto,2003 study, mammograms were 2-view in the first round and were 1-view in subsequent rounds. For the No Author, 1999 study, type of mammogram varied by screening center. Women in the screened group also received clinical breast exam during non-screened year.

D. Study authors reported all mortality and population numbers by age categories. We excluded women screened ages 40–49 because of the overlap with the Hellquist paper. We then separated the data for women screened 50–69 and 70–74 in order to include in meta-analyses for each age category.

E. RR and CI reported in this table are only for the comparison of Gavleborg to all of Sweden.

F. RR and CI reported in this table are only for the comparison of Turku to Helsinki. The comparison of Turku to Tampere was not included because of overlap and because Tampere screened women 55–59.

G. RR slightly different as RR reported in original article was adjusted for pre-trial rates, which we did not do.

H. Study only reports aggregate population data. No individual cases are identified or linked from medical records or cancer registries.

I. The specific year that screening began varied by county or region.

J. Screening expanded to women age 70 in 2002.

K. We only analyzed women in cohort 1.

Studies did not always report the same metric; equivalent metrics were calculated to compare between the studies. For each study, the following items were calculated: relative risk, population (number of women) and person-years (number of women times number of years of follow-up). If person-years (or population) were not provided, they were calculated by multiplying (or dividing) the average years of study period by the average annual population (or person-years). If a relative risk and confidence interval were not provided, they were calculated from the number of cases and person-years. Relative risk and confidence intervals for geographical-historical studies were calculated using procedures detailed in Altman and Bland [35].

Meta-analyses were conducted in Stata MP Version 12 (StataCorp, College Station, Texas) using the metan command, random effects. Random effects model was performed because statistical heterogeneity existed. Birth cohorts, historical, geographical and geographical-historical designs were analyzed and reported separately. Within each design, a separate meta-analysis was conducted for each screening age range (<50, 50–69, 70+). Meta-analyses were conducted using the standard weighting procedure (standard error of the studies) and then weighted by total population or total person-years. The resulting RR and CI for each design, screening age and weighting strategy are reported in Table 2. Incident and prevalent breast cancer mortality are analyzed separately. Assessment of bias was analyzed as threats to validity. Each study design was scored according to potential threats to internal and external validity (Table 3).

**Table 2:** Summary of meta-analysis results by design and age screened^a^.

Design & Age Group Screened	Number of Studies	RR (95% CI) BC Mortality	RR (95% CI) BC Mortality Adjusted for Population Size	RR (95% CI) BC Mortality Adjusted for Person-Years
**Study design: Birth cohorts**				
Incidence-based breast cancer mortality				
Screened ages <50	1	0.11 (0.01, 0.85)	0.11 (0.01, 0.85)	0.11 (0.01, 0.85)
Screened ages 50–59	2	0.77 (0.57, 1.03)	0.77 (0.57, 1.03)	0.77 (0.60, 1.00)
**Study design: Historical Comparisons**				
Incidence-based breast cancer mortality				
Screened ages 40–69	2	0.67 (0.54, 0.82)	0.57 (0.44, 0.74)	0.57 (0.44, 0.74)
Prevalence-based breast cancer mortality				
Screened ages40–69	3	0.79 (0.62, 0.99)	0.77 (0.76, 0.78)	0.76 (0.75, 0.77)
**Study design: Geographical Comparison**				
Incidence-based breast cancer mortality				
Screened ages <50	2	0.80 (0.73,0.88)	0.79 (0.73,0 .87)	0.79 (0.73,0.87)
Screened ages 50–69	2	0.78 (0.70, 0.87)	0.79 (0.70, 0.88)	0.78 (0.71, 0.87)
Screened ages 65+	2	0.88 (0.65, 1.19)	0.92 (0.65, 1.29)	0.92 (0.65, 1.31)
**Study design: Geographical-Historical Comparisons**				
Incidence-based breast cancer mortality				
Screened ages <50	1	0.91 (0.72, 1.15)	0.91 (0.72, 1.15)	0.91 (0.72, 1.15)
Screened ages 50–69	5	0.83 (0.72, 0.95)	0.84 (0.76, 0.94)	0.87 (0.78, 0.97)
Screened ages 70+	1	0.97 (0.74, 1.28)	0.97 (0.74, 1.28)	0.97 (0.74, 1.28)

a. Each cell represents the RR and 9% CI for a separate meta- computed using the metan random effects model in Stata. The historical comparisons included some studies with prevalence-based breast cancer. For historical designs, both incidence and prevalence-based breast cancer are reported but in separate rows. Incidence-based breast cancer excludes women diagnosed with breast cancer before the screening program was initiated. Prevalence-based breast cancer does not.

**Table 3:** Catalogue of potential threats to internal and external validity of breast cancer screening quasi-experimental studies stratified by specific design^a^.

	Birth Cohort (n = 3)	Historical (n = 4)	Geographical (n = 5)	Historical by Geographical (n = 7)
Number of studies that analyzed only aggregate data	0	2	0	0
Death data ascertainment				
National or regional death, health or cancer registries	3	3	3	7
Other	0	0	2^c^	0
Not reported	0	1^b^	0	0
Threats to internal validity				
Maturation	No	No	No	No
Attrition	No	No	No	No
Testing	Yes	Yes	Yes	Yes
History	No	Yes	Possible	No
Instrumentation	No	Yes	Possible	Possible
Regression	No	Possible	Possible	No
Selection	No	No	Possible	Possible
Interaction of selection by maturation	No	No	No	No
Threats to external validity				
Interaction of testing by screening program	Yes	Yes	Yes	Yes
Interaction of selection by screening program	No	Yes	Yes	Yes
Interaction of setting/history by screening program	Possible	Yes	Yes	Possible
Multiple-Program Interference	No	Yes	Possible	Possible

a. List and definitions of threats of validity from Grembowski D. The Practice of Health Program Evaluation. Sage Publications: Thousand Oaks, CA; 2001.

b. Study did not report the source of death data but it was assumed that they came from national health system and registries from the UK.

c. Other forms of death verification: regional radiology departments, carcinoma working groups and panels of physicians.

## Results

The search strategies returned 4,903 abstracts of which 116 full-text articles were reviewed (Figure 1). A total of 19 studies were retained in the final analyses (See Table 1). All studies retained in this review implemented screening programs using mammograms, reported breast cancer mortality, and were available in English.

Table 1 displays the descriptive details for the studies included in the meta-analysis. All studies were from European countries with a national healthcare system – England, Finland, the Netherlands, Denmark Norway, Spain and Sweden. Years of publication ranged from 1995–2013. The majority of studies had screening intervals of 2 years and analyzed programs which screened women ages 50–69. About two-thirds of studies reported attendance rates and these ranged from 46–90%. Study years ranged from 5–22 years, although individual women were not necessarily followed the entire length of the study. Only two studies had follow-up periods less than 10 years. Some studies were not able to report the average length of follow-up per individual women because they analyzed aggregated population data, not individual data. The studies varied in the ascertainment of cause of death. Sixteen studies received their cause of death from national or regional death or cancer registries, 2 studies from the regional radiology departments, and 1 study did not state how they ascertained cause of death.

A funnel plot was constructed to assess publication bias. Figure S1 displays a funnel plot for the relative risks of the 19 quasi-experimental studies analyzed in this review. All age groups screened were included in the funnel plot. The funnel plot is symmetrical suggesting that there is no publication bias among these quasi-experimental studies. One study is shown as an outlier with a strong beneficial relative risk [36]. The results of this study are discussed in the outcomes for the birth cohort comparisons, below.

### Birth cohort comparisons

Finland gradually implemented their screening program by inviting specific birth cohorts to screening and compared their morality to non-screened birth cohorts. For example, women born in 1936 were invited to screening while women born in 1937 were not [24], [36]. In the study by Antilla et al, authors compared the cohort of women born in 1930–1934 who were never screened to the cohort of women born in 1935–1939 who were offered screening [1]. The three birth cohort studies were all from Finland and screened different ages (Table S3) [1], [24], [36]. Two studies which screened women ages 50–59 showed non-significant reductions in incident breast cancer mortality between 20–24% depending on end date of follow-up [1], [24]. Their pooled RR was non-significant (RR = 0.77 with 95% CI (0.60, 1.00)). For the one study which screened women below age 50, the screening program showed a significant 89% reduction (RR = 0.11 (0.01 0.85)) in incident breast cancer mortality between birth cohorts [36].

### Historical comparisons

These studies compared a distinct geographical region before and after implementation of a population screening program. Four historical studies were identified in four different countries –England, the Netherlands, Spain, and Sweden [3], [28], [37], [38]. All areas screened women ages 50–65. In Sweden and Spain, women as young as age 40 and 45, respectively, were invited to screening. However, the studies did not separate out breast cancer mortality for women <50.

There were significant 3 to 43% reductions in breast cancer mortality as compared to the reference period in the individual four studies (Table S4). Only one of the studies analyzed screening and reference periods of equal length (10 years each) with no gap in time between the end of the reference period and start of the screening period [38] The other studies compared reference and study periods that were not equal in length and allowed a lag time of 5–6 years between the end of the reference period and start of the screening period [3], [28], [37]. Studies that allowed for a lag time between implementation of screening and measurement of breast cancer mortality reported a larger, protective benefit associated with screening than studies that measured mortality from the start of the program.

These significant reductions in breast cancer mortality were replicated in the meta-analysis whether weighted by the standard error, population size or person-years (Table 2). Studies that provided incidence-based breast cancer mortality reported stronger, protective benefits of screening (RR = 0.57 (0.44, 0.74) n = 2) than studies providing prevalent-based breast cancer mortality (RR = 0.76 (0.75, 0.77) n = 3), although both were statistically significantly.

### Geographical comparisons

These studies compared region(s) within a country which implemented a screening program to others areas within the same country without a program. Both screening and non-screening regions had equal access to treatment for breast cancer. Five geographic comparisons were found −2 from the Netherlands, 2 from Sweden, and 1 from England [31], [39]–[42]. Jonssson et al., 2007 screened women 40–74 and provided separate mortality numbers for women 40–49, 50–69, and 70+. Because of overlap with the study by Hellquist et al, 2011 included in this review, data for women 40–49 from Jonsson et al., 2007 were excluded; data for the effects for women 50–69 and 70+ were retained and analyzed as unique studies analyzing the separate age categories [39], [40]. All studies reported incident breast cancer mortality.

Meta-analyses of the 2 studies for women screened below age 50 produced a significant reduction in breast cancer mortality with screening weighted either by population or person-years (RR =  .79 and 95% CI (.73, .87)) [39], [42]. See Table 2 and Table S5. As independent studies, only Hellquist et al., 2011 showed a significant benefit to breast cancer mortality (RR = 0.79 (0.72, 0.86)) while Peer et al., 1995 did not (RR = 0.94 (0.68, 1.29)) [39], [42]. Two geographic studies analyzed breast cancer mortality for screening for women 50–69. Their pooled effects showed significant protective mortality benefits for women screened ages 50–69 (RR =  .78 and 95% CI (.71, .87)) [40], [41]. Two studies provided mortality data for women screened ages 68+ and did not show a significant mortality benefit for mammography as independent studies or when pooled [31], [40].

### Geographical-Historical Hybrids

Geographical-historical hybrids are designs that can test the interaction of geographical and historical studies. The RR of the screening region pre-post screening implementation is compared to the RR of the non-screening region during the same time periods. There were 7 geographical-historical hybrid comparisons, 4 were from Sweden, 1 from the Denmark, 1 from Norway and 1 from Finland [30], [33], [43]–[47]. All studies reported incident breast cancer mortality. See Table 2 and Table S6.

Only Jonsson et al.,2000 analyzed screening for women under 50 years of age and only Jonsson et al., 2003 analyzed the effects for women over 67. The results were non-significant for both (Table 2) [41], [43]. Neither could be pooled because there was only an N = 1 within each age category. There were 5 geographic-historical hybrids that screened women 50–69 (or 40–64 for the Jonsson et al., 2003 as the effects for women 50–69 could not be disentangled) [30], [33], [46], [44], [47]. As independent studies, only Olsen et al., 2005 and Parvinen et al., 2006 reported significant reduction in breast cancer [30], [47]. When the data are pooled for all geographical-historical hybrids, there was a significantly protective benefit of 13–17% whether weighted for population or person-years.

### Comparing biases across study designs

The RCT meta-analyses ranked studies by their fidelity to randomization [7], [8]. Since we did not include RCTs, we compared studies by design and by threats to validity. Table 3 lists possible threats to internal and external validity for each of the four study designs. In addition, Table 3 codes whether the study designs included individual data from women or only aggregate population data and how death data was ascertained. Threats to internal validity include: maturation, attrition, history, testing, instrumentation, regression, selection, and the interaction of selection by maturation. Threats to external validity include the interactions of the program by testing, selection, setting/history as well as interference from multiple programs. These threats were originally defined by Campbell and Stanley (1966) and adapted for health studies by Grembowski (2001) [48], [49]. Study designs were code as having a threat to validity – yes, no or possible. For studies coded as yes, this threat to validity would be expected in most or all cases. For studies coded as possible, this threat to validity could occur in some scenarios although it seems unlikely.

Maturation and attrition were not coded as threats to internal validity in any of the designs. A threat to validity due to maturation suggests that a woman's response to the program or evaluation would be altered because of her aging process. Maturation was not coded as a threat because all designs included control groups of woman of the same age and would be expected to mature at approximately the same rate. Attrition is minimized in all of these designs because women were not selected or self-selected for participation. These studies either 1) used aggregate data only or 2) applied the intervention to all women living in a region, time period or birth year and monitored their breast cancer diagnosis or death through national or regional registries, or local radiology departments.

We scored all the studies as having a threat to internal and external validity due to testing of a screening program. In the case of screening mammography, the test (or the screening mammogram) is not given in both groups. If a woman has a positive mammogram but turns out to not have cancer, she may experience additional anxiety or unnecessary follow-up procedures.

For threat to internal validity due to changes in history, we coded historical comparisons as a yes and geographical comparisons as a possible. Historical comparisons are unable to control for improvements in cancer treatment or heightened breast cancer awareness that would have occurred over time. Women in the screening time periods would be more aware of breast cancer, might take more notice of breast abnormalities and might be more interested in being screened. For the geographical comparisons, studies do not analyze pre-screening rates to determine if there are differences between the regions on historical trajectories of breast cancer mortality or if there is a difference on the age distributions between the regions.

Threats to instrumentation might arise because of technical improvements in the sensitivity of mammography and refined skills of radiologists and pathologists. The historical comparison designs definitely have this threat because instrumentation should have improved over many decades. Geographical or geographical-historical interactions have a possible threat to internal validity from instrumentation if the same types and models of equipment were not used in the control regions.

Regression effects might occur if a program is implemented in an area where rates are artificially high. For example, a community might start screening for breast cancer in response to high breast cancer rates. A reduction in breast cancer in that community could be attributable to screening, but it would not rule out the alternative explanation of a regression effect. We scored historical and geographical comparisons as having a possible validity threat due to regression to the mean. For historical comparisons, breast cancer deaths might already have been declining due better treatment or other trends. Geographical comparisons cannot rule out temporal trends. There might be differences in baseline rate of breast cancer diagnosis and death between each region that are unaccounted for in the geographical designs. Regression to mean is not a threat to the designs that included the interaction of geographical-historical because the design controls for baseline rates and trajectories that might differ between regions.

Geographical comparisons & geographical-historical hybrids might have possible selection biases as compared to the other designs. In these designs, women in one region(s) were assigned to screening while other region(s) were used as a control. These studies could not rule out heightened awareness, an increase of breast cancer specialists in that region, increased social interactions with women in the region who had experience with screening or subject differences between regions. There might be differences between these regions aside from just having a screening program (i.e. socio-economic differences, rural vs urban, value placed on preventive health care, age distribution differences between the studied populations). For instance, the study in the UK mentioned that screening regions also included clinician breast exams with the screen and offered open-access clinics to women if they detected any abnormalities [41].

For threats to external validity, all designs have a possible threat due to program by testing effect. Because there is a threat to internal validity due to testing, we felt all designs would also have a threat to external validity. External validity may be threatened by the interaction of the program by selection for three of the study designs –historical, geographical, and historical-geographical interactions. The screening regions were picked and were started in a certain order for non-random reasons. Policymakers selected the screened cities or counties presumably for reasons that would lead to the success of the program. Possible examples could be: citizens with more favorable attitudes towards health and prevention; regions with more resources to quickly set-up a population-level program; or increased needs such as higher baseline rates of breast cancer. All designs would have a possible or definite threat to external validity to the interaction of the program by setting/history. These same findings might not be observed if replicated in settings with different medical care settings or more heterogeneous populations.

Multiple program interference is a definite threat in historical designs because of other breast cancer awareness programs, such as mass media campaigns and the increase in private screening. Geographical and geographical-historical interactions might have possible threats to multiple program interference because of changes in the social environment of screened areas. Women in regions with screening would be more likely to talk about screening and how screening saved their lives from breast cancer.

Historical designs were coded as having the most threats to both internal and external validity, followed by geographical and then geographical-historical studies. We scored birth cohort studies as having the fewest threats to validity. However, this design was only implemented in Finland and these results may not work in a more heterogeneous population or a population without universal health care and linked medical records.

## Discussion

Although debate about the design and execution of the RCTs versus quasi-experimental trials continues, no new RCTs that will definitively inform the debate are expected to be published in the foreseeable future. Reports of longer term follow-up of prior study participants are informative but may not settle the controversy [50]. In addition, RCTs are often criticized because they create artificial service delivery models and use participants that are not representative of the populations to whom the results will be generalized [16], [17].

Quasi-experiments may not have the internal validity of RCTs, but often deliver interventions in real practice settings using representative populations. They are also subject to biases, such as opportunistic screening. When Norway started a population screening program, 40% of Norwegian women had already undergone a mammogram prior to their first invitation to the population program [51]. Other concerns raised about quasi-experimental designs are the short-term follow-up and potential for publication bias. Almost all of our quasi-experimental designs had 10 years or more of follow-up post screening. However, most of the studies continued to accrue women (continued to screen new women in their study population) over the follow-up period. Women who received their first screen later in the accrual period may not have had sufficient years of follow-up and this effect may have under-estimated the benefit attributed to screening. The number of years of follow-up for an individual would have been less than 10 for most of the study women. Longer term follow-up in RCTs have not revealed a stronger effect of screening [50] and we expect similar results for quasi-experimental studies. There may be concern that quasi-experimental studies with null results would be less likely to be published than those with favorable results. The funnel plot (Figure S1) suggests that publication bias among these quasi-experimental studies is not likely.

Findings from our meta-analyses of quasi-experimental studies paralleled findings from the meta-analyses of RCTs. Mammography screening was beneficial for women screened age 50 to 69. The results were mixed for women screened under age 50 and null for women screened at age 70 and older.

The RCT meta-analyses ranked studies by their fidelity to randomization [7], [8]. Since we did not include RCTs, we compared studies by design and by expected level of validity [48], [49]. The pooled effects sizes varied depending on type of study design. Larger effect sizes were observed in designs with lower levels of expected validity (i.e. historical comparisons); smaller effect sizes were observed in designs with higher levels of expected validity (birth cohorts & geographical-historical hybrids). The strongest study design was the birth cohort design. However, this design was only implemented in Finland and there is some threat to generalizability because it was not applied in heterogeneous populations. Geographical-historical interactions are the next strongest design and were tested with data from four different counties. Although most of the threats to internal validity for geographical studies still apply to the geographical-historical interactions, these interaction designs can control for the underlying temporal change in breast cancer screening outcomes.

### Women screened under age 50

For women screened under age 50, results of the RCT meta-analyses were mixed with some studies reporting benefits of screening [7], [8]. In the quasi-experimental meta-analysis, there also were mixed findings among the 4 studies that screened women under age 50. Geographical comparisons showed a significant reduction in breast cancer mortality for women screened under age 50, but this pooled effect was primarily attributed to one study [39]. The birth cohort study that screened women under 50 appears to be an outlier in the funnel plot shown in Figure S1 (RR = 0.11). The study authors concluded that the 89% reduction in breast cancer mortality could not be attributed solely to screening [36]. The geographical-historical hybrid (n = 1) reported null findings for women screened below age 50 [43].

### Women screened over age 70

Across all designs, there were only three studies that screened older women (68+) [31], [40], [46]. None found an effect of screening on breast cancer mortality as independent studies or when pooled. Our results are consistent with the null findings in the meta-analyses of RCTs [8].

### Women screened age 50–69

Across studies, there was a significant decrease in breast cancer mortality for women screened between ages 50–69; however the effect depended on which studies were included. In the meta-analyses, the birth cohort studies for women screened ages to 50–69 showed a non-significant reduction in breast cancer mortality. Historical comparison studies found significant effects independently and produced a pooled 33–43% risk reduction in incident breast cancer mortality and a 21–24% reduction in prevalent breast cancer mortality. However, historical designs have limited validity because they are unable to control for improvements in cancer treatment or heightened breast cancer awareness. The meta-analyses of geographical comparisons produced a similar benefit of approximately 22% relative risk reduction of breast cancer mortality. Geographical studies could not rule out heightened awareness, an increase of breast cancer specialists in that region, or subject differences between groups.

The geographical-historical hybrids could rule out temporal and between group differences. There were a sufficient number of studies for women screened 50–69. There was a significant 13–17% reduction in breast cancer mortality, even though the majority of studies did not observe significant results as independent studies. These effects are tempered when one considers the large number of women in population studies (over 8,000,000 person-years in the exposed cohorts when pooled across geographical-historical studies). The pooled relative risk reduction seen for these studies matches the meta-analysis results for RCTs (15% RR reduction) [7].

### Alternate explanations

Other studies have suggested that breast cancer survival has been improving over time because of factors other than screening [52], [53]. Sun and colleagues attempted to separate the effects of cancer screening from the improved cancer treatments [52]. Using SEER data, they estimated benefits of screening from rates of early detection. Benefits of improved treatments were estimated from changes in state-conditional survival. They concluded that between 1988 and 2000 improvements in breast cancer survival owed more to improved treatment than to screening [52]. Thus, advances in cancer treatment cannot be ruled out as an alternative explanation for the benefits observed.

### Comparisons with other reviews

Two recent meta-analyses reported a 25% pooled relative risk breast cancer mortality reduction for geographical-historical hybrids [22], [23]. As with any review, the effect sizes can shift with the inclusion or exclusion of certain studies. The selection of studies in this meta-analyses differed from their selection of studies by just a few studies. We performed sensitivity analysis by excluding or adding different studies, but we did not reach a RR of 0.75 that was reported in these studies. The Njor et al., 2012 review included Jonsson et al, 2007 as a geographical-historical hybrid; this review considered it a geographical comparison because population sizes or person-years were not available for every period [22]. If treated as a geographical-historical hybrid, the Jonsson et al., 2007 independent RR would have been 0.86 (0.63, 1.17) which would have matched pooled findings in this paper. Both Broeders et al., 2012 and Njor et al., 2012 included a study by Sarkeala et al., 2008, which this analysis excluded because it compared observed to expected breast cancer mortality [22 22, 54]. The Sarkeala et al. 2008 paper does provide observed mortality in screened and control groups, but these numbers compared women within the same region who self-selected into screening versus those who were not screened or compared of women invited regularly or irregularly over the age of 60. Neither of these comparisons matched our inclusion criteria or research question [52]. The Sarkeala et al., 2008 study reported a significant RR = 0.69 (a 31% reduction in breast cancer mortality) which contributed to the larger, protective finding in the Njor & Broerders, 2012 studies. Lastly, although the Njor et al. 2012 paper identified similar studies as in this review, they did not pool all studies in their meta-analysis and produced different pooled RR than our analyses. It is important to have a clear understanding of the pooled relative risks from meta-analyses because these estimates are often incorporated into simulation models and used in planning screening programs.

### Limitations

Conclusions from this paper are limited for several reasons. First, although we believe our searches were comprehensive, we only identified studies of European screening programs, with presumably primarily Caucasian samples. One publication from Russia was excluded because it only analyzed two years post-implementation of the program [55]. We also found a limited number of studies, although this number of studies in the analysis is comparable to other systematic reviews of observational studies and RCTs for breast cancer screening [7], [8], [18]–[23]. We believe our search was exhaustive of the published studies meeting our criteria.

No studies from the United States were included because none could compare a region or time period with an official screening program. Studies conducted with U.S. data compared regions with higher rates of screening to those with lower rates of screening. States with higher rates of mammography tend to have a lower all-cause, 2-year case fatality rate although this data was restricted to Whites receiving Medicare (typically age 65+) [56]. Further, these states also tend to have better health outcomes for conditions unrelated to cancer. NCI's Historical Connecticut Tumor Registry reported that breast cancer mortality rate declined 31.6%, but mortality fell for women too young for routine screening and rates declined more slowly for late-stage disease incidence suggesting some improvement in mortality not attributed to early detection [57].

Second, all-cause mortality could not be analyzed because there were an insufficient number of studies reporting this data (only 2 studies). Olsen et al, 2005, reported that breast cancer screening did not reduce all-cause mortality [58]. Tabar et al., 2003, compared women in regions with a screening program versus women in regions without a screening program and reported significant benefits in all-cancer and all-cause mortality [27]. Although meta-analysis of RCTs also suggests benefits for breast cancer mortality, the aggregated studies failed to show that screening increases life expectancy, as evidenced by analysis of all-cause mortality [7], [8].

Third, our review was not registered because we were unaware of registration services when our effort began. Registration reduces biases, promotes transparency of methods and avoids potential duplication. In order to demonstrate transparency and limit bias, we provided our search strategies, number of results returned and excluded, and raw numbers and RR used in calculations (all available in online supplementary materials). Replication is essential to all studies, even meta-analyses and we encourage others to replicate our findings. One of the main concerns with meta-analysis is the selection of which studies to include and which to exclude. Replication of meta-analyses can highlight what happens when certain studies are excluded or included. In our methods section, we detailed which studies we excluded because they overlapped with included studies. We conducted subsequent meta-analyses replacing the included study with the study that was excluded because of overlap. These substitutions produced similar results. For the birth cohort analyses, if we replaced the six-year follow-up data with the nine-year follow-up data, the pooled RR for birth cohort studies among women 50–69 was still non-significant (RR = 0.95 with 95% CI (0.77, 1.17)) [24], [25]. In the geographical analyses, we analyzed Van Dijick et al, 1997 instead of Broeders et al, 2001, but both studies reported non-significant, reduction in breast cancer mortality [31], [32]. In the geographical-historical hybrids, we substituted the Jorgensen et al., 2010 and Kalager et al., 2010 studies in place of the Olsen 2005 and 2013 studies [29], [30], [33], [34]. The protective benefit for screening among women 50 to 69 was reduced to 11–12%, but still remained significant.

Lastly, we re-calculated relative risks and confidence intervals for each of the studies to include them in the meta-analysis. Our calculations were almost always similar to the original study outcomes. However, the calculation of the RR may not have been appropriate for some of the designs and may have led to alternate conclusions. Jorgensen et al., 2010 reported null findings for screening women ages 55–74 [29]. They used Poisson regression analyses and quantified a 1% breast cancer mortality reduction per year in screened areas and a 2% breast cancer mortality reduction per year in non-screened areas. Furthermore, in women too young for population screening, they calculated a 5% reduction in screened areas and 6% reduction in non-screened areas per year. In order to include this study in the meta-analyses, the RR was re-calculated using methodology similar to the other geographical-historical hybrids with data provided in the original publication. The re-calculated RR showed a 0.84 RR (0.76, 0.95) if a 5 year lag time between implementation of screening and measurement was included. If no lag time, there was a non-significant effect (RR = 1.07).

## Conclusions

Future studies are needed, especially analyzing programs that screen women under 50 or over 70 years of age. Quasi-experimental studies often do not report study details to the same extent as RCTs which made it difficult to determine whether to include the quasi-experimental study in the analyses. In the future, all studies should report the population number and person-years for screened and unscreened cohorts. Raw mortality numbers should be included, not just adjusted RRs or trend lines.

Overall, our meta-analyses of the geographical-historical studies showed a significant reduction in breast cancer mortality of 13–17% attributed to screening when adjusted for person-years. The analyses did not adjust for lead time, attendance, or self-referral because not all the studies included these adjustments. Several of the quasi-experimental studies only showed significant differences once adjusted for these factors or once a lead time of 5–10 years was incorporated. Stronger reductions in breast cancer mortality were observed when a gap or lag time was allowed between the start of the screening program and the start of measurement.

In summary, new RCTs are not likely to inform the controversy over the value of breast cancer screening in the near future. Quasi-experiments or natural experiments may often use more representative study populations than RCTs. Although results vary across studies, in aggregate there is a benefit of screening women 50–69 years of age.

## Supporting Information

Figure S1 Funnel plot of relative risk of breast cancer mortality from all quasi-experimental studies that compared a region with a screening program to a region without a screening program. This funnel plot includes all studies with various designs, age groups and both incidence or prevalence breast cancer mortality.
(EPS)

Checklist S1 PRISMA 2009 Checklist.
(DOC)

Table S1 Search strategies in Pub Med and Embase and Abstracts Returned.
(DOC)

Table S2 Review of Pub Med and Embase Articles: Reasons abstracts excluded from review.
(DOC)

Table S3 Data extracted from birth cohort comparisons of breast cancer screening programs.
(DOC)

Table S4 Data extracted from historical comparisons of breast cancer screening programs.
(DOC)

Table S5 Data extracted from geographical comparisons of breast cancer screening programs.
(DOC)

Table S6 Data extracted from geographical-historical interaction designs of breast cancer screening programs.
(DOC)
