# Supplementary material for: Screening Mammography & Breast Cancer Mortality: Meta-Analysis of Quasi-Experimental Studies
Source: PLoS One. 2014 Jun 2;9(6):e98105. doi: 10.1371/journal.pone.0098105 (PMC4041743; doi:10.1371/journal.pone.0098105)
Supplement: Table S1 — Search strategies in Pub Med and Embase and Abstracts Returned. (DOC) [file pone.0098105.s003.doc]

| **Table S1. Search strategies in Pub Med and Embase and Abstracts Returned** | | |
| --- | --- | --- |
| MeSH, Emtree headings, or Title and Abstract | **Pub Med** | **Embase** |
| 1. Breast Neoplasm OR breast | 283,880 | 296,955 |
| 2. Early Detection of Cancers OR Mammography OR Mass Screening | 107,125 | 214,204 |
| 3. Mortality OR Survival analys* OR survival rate | 661,371 | 924,096 |
| 4. Combined searches AND; plus limit to humans | 2,937 | 5,8502 |
| 5. NOT BRCA1, BRCA2 Protein/genetics; DNA; Genes | 2,904 | 5,495 |
| 6. NOT chemotherapy; radiotherapy | 2,762 | 5,391 |
| 7. NOT Health, Knowledge, Attitudes & Practice | 2,642 | 5,353 |
| 8. NOT ultrasonography; radiology; Tumor Markers | 2,583 | 5,329 |
| 9. NOT radiation dosage | 2,546 | 5,268 |
| 10. NOT publication – editorial, letter, comment | 2,195 | 3,635 |
| 11. Remove Duplicates | 2,188 | 3,612 |
| 12. Remove duplicates with PubMed | 2,188 | 2,481 |
| 13. Updates through January 31, 2013 | 61 | 173 |
| Total abstracts reviewed per database | 2,249 | 2,654 |
