# Supplementary material for: Screening Mammography & Breast Cancer Mortality: Meta-Analysis of Quasi-Experimental Studies
Source: PLoS One. 2014 Jun 2;9(6):e98105. doi: 10.1371/journal.pone.0098105 (PMC4041743; doi:10.1371/journal.pone.0098105)
Supplement: Table S2 — Review of Pub Med and Embase Articles: Reasons abstracts excluded from review. (DOC) [file pone.0098105.s004.doc]

| **Table S2. Review of Pub Med and Embase Articles: Reasons abstracts excluded from review** | | |
| --- | --- | --- |
|  | **PubMed (Medline)** | **Unique Embase* (Excluding Medline)** |
| No Abstract | 92 | 133 |
| Full articles read | 78 | 38 |
| No (specific exclusions listed below) | 2,079 | 2,483 |
| TOTAL | 2,249 | 2,654 |
|  |  |  |
| Exclusions: |  |  |
| Editorial/Comment; non-systemic review; no original data | 576 | 563 |
| Correlations only | 76 | 143 |
| No comparison group, trends, joinpoint regressions | 230 | 113 |
| Qualitative | 14 | 12 |
| Wrong “intervention” (inc screening, work with clinicians) | 80 | 92 |
| Meta-analysis, review | 90 | 96 |
| Diagnostic, assessment tools, genetics, biomarkers | 112 | 253 |
| Treatment | 26 | 246 |
| Simulation or mathematical, cost-effectiveness | 122 | 76 |
| Random control trial | 73 | 14 |
| Wrong population (women already with breast cancer; women with family history) | 201 | 315 |
| No mortality | 180 | 28 |
| Wrong cancer, disease, multiple cancers | 150 | 266 |
| Policies/recap for state, agencies, or conference and societies | 95 | 110 |
| Case control, case-referent | 39 | 21 |
| Not relevant | 7 | 62 |
| Earlier - full trial published later & included | 4 | 0 |
| Translation of previous study | 1 | 0 |
| Animal study | 0 | 2 |
| Case study(ies) | 0 | 68 |
| News/media coverage | 3 | 1 |
| Language | 0 | 1 |
| Duplicate | 0 | 1 |
| *Embase returns that were not found in Medline search. | | |
