# Supplementary material for: Screening Mammography & Breast Cancer Mortality: Meta-Analysis of Quasi-Experimental Studies
Source: PLoS One. 2014 Jun 2;9(6):e98105. doi: 10.1371/journal.pone.0098105 (PMC4041743; doi:10.1371/journal.pone.0098105)
Supplement: Table S3 — Data extracted from birth cohort comparisons of breast cancer screening programs. (DOC) [file pone.0098105.s005.doc]

| **Table S3. Data extracted from birth cohort comparisons of breast cancer screening programs** | | | | | | | | |
| --- | --- | --- | --- | --- | --- | --- | --- | --- |
|  | Non-screened birth cohorts | | | | Screened birth cohorts | | | |
|  | Average # study years | # of breast cancer deaths^a^ | Sample population | Person-years^b^ | # of breast cancer deaths^a^ | Sample population | Person-years^b^ | Re-calculated RR (95% CI)^c^ |
| **Screened ages <50** | | | | | | | | |
| Hakama, 1995 | 10 | 13 | 6,223 | 62,230 | 1 | 4,319 | 43,190 | 0.11 (0.01,0.85) |
| **Screened ages 50-69** | | | | | | | | |
| Anttila, 2002 | 10 | 71 | 15,540 | 186, 480 | 59 | 16,140 | 193,680 | 0.80 (0.57, 1.13) |
| Hakama, 1997 | 6 | 63 | 68,862 | 299,228 | 64 | 89,893 | 400,804 | 0.76 (0.54, 1.07) |
| 1. Deaths represent incidence-based breast cancer mortality. 2. Person-years not provided in any study. We calculated by multiplying population by study years. 3. Relative risk calculated from number of breast cancer deaths and person-years. | | | | | | | | |
