# Supplementary material for: Screening Mammography & Breast Cancer Mortality: Meta-Analysis of Quasi-Experimental Studies
Source: PLoS One. 2014 Jun 2;9(6):e98105. doi: 10.1371/journal.pone.0098105 (PMC4041743; doi:10.1371/journal.pone.0098105)
Supplement: Table S4 — Data extracted from historical comparisons of breast cancer screening programs. (DOC) [file pone.0098105.s006.doc]

| **Table S4. Data extracted from historical comparisons of breast cancer screening programs** | | | | | | | | | | |
| --- | --- | --- | --- | --- | --- | --- | --- | --- | --- | --- |
|  |  | Reference Period (Pre-screening) | | | | Study Period (Post Screening | | | | |
| Author, year of publication | Ages Screened | Average # study years | # Deaths | Person-Years | Average Annual Population | Average # study years | # Deaths | Person-Years | Average Annual Population | Re-calculated RR (95% CI)^a^ |
| **Prevalence-based breast cancer mortality** | | | | | | | | | | |
| Ascunce, 2007^b,c^ | 45-69 | 5 | 185 | 289,063 | 57,813 | 6 | 123 | 296,386 | 59,277 | 0.65 (0.52, 0.81) |
| Duffy, 2010 ^b,c^ | 50-69 | 15 | 77,805 | 79,604,000 | 5,306,933 | 10 | 38,201 | 53,427,972 | 5,342,797 | 0.73 (0.72, 0.74) |
| Otto, 2003^d^ | 50-69 | 10 | 14,971 | 14,252,827 | 1,425,283 | 10 | 8,414 | 8,227,529 | 822,753 | 0.97 (0.95,1.00) |
| **Incidence-based breast cancer mortality** | | | | | | | | | | |
| Ascunce, 2007 ^b,c^ | 45-65 | 5 | 150 | 289,063 | 57,813 | 6 | 88 | 296,386 | 59,277 | 0.57 (0.44, 0.74) |
| SOSSEG, 2006 | 40-69 | __e | 2,736 | 7,265,841 | 542,187 | __e | 2,042 | 7,542,833 | 566,423 | 0.72 (0.68, 0.76) |
| 1. Relative risk calculated from number of breast cancer deaths and person-years. 2. Either population or person-years were not provided in the original article, but were re-calculated from number of breast cancer deaths, mortality rates and other population parameters. 3. These two trials waited 5 – 6 years after the start of the screening program before beginning to analyze breast cancer mortality data. 4. Relative risk ratios were not reported in the original article. We re-calculated the RR and 95% CI using data found in Table 3 of the original article. 5. Study years varied depending on area and ranged from 10-22 years. Study authors calculated person-years and population estimates for each area. | | | | | | | | | | |
