# Supplementary material for: Screening Mammography & Breast Cancer Mortality: Meta-Analysis of Quasi-Experimental Studies
Source: PLoS One. 2014 Jun 2;9(6):e98105. doi: 10.1371/journal.pone.0098105 (PMC4041743; doi:10.1371/journal.pone.0098105)
Supplement: Table S5 — Data extracted from geographical comparisons of breast cancer screening programs. (DOC) [file pone.0098105.s007.doc]

| **Table S5. Data extracted from geographical comparisons of breast cancer screening programs** | | | | | | | | | | | |
| --- | --- | --- | --- | --- | --- | --- | --- | --- | --- | --- | --- |
|  |  | Reference Group (No screening) | | | | Study Group (Screening) | | | | |  |
| Author, Year of publication | Ages Screened | Average # study years | # Breast Cancer Deaths^a^ | Person-Years | Average Annual Population | Average # study years | # Breast Cancer Deaths^a^ | Person-Years | Average Annual Population | | Re-calculated RR (95% CI)^b^ |
| **Screened ages <50** | | | | | | | | | | | |
| Hellquist, 2002 | 40-49 | 15.7 | 1,238 | 8,843,852 | 567,538 | 15.8 | 803 | 7,261,415 | 459,977 | | 0.79 (0.72, 0.86) |
| Peer, 1995 | 35-49 | 16 | 74 | 154,103 | 9,631 | 16 | 75 | 166,307 | 10,394 | | 0.94 (0.68, 1.30) |
| **Screened ages 50-69** | | | | | | | | | | | |
| Jonsson, 2007^c^ | 50-69 | 18 | 155 | 539,184 | 41,322 | 18 | 163 | 707,742 | 59,109 | | 0.80 (0.64,1.00) |
| No Authors, TEDBC, 1999^d^ | 45-64 | 14.7 | 1,312 | 1,871,852 | 127,123 | 14.5 | 360 | 661,711 | 45,607 | | 0.78 (0.69, 0.87) |
| **Screened ages 67+** | | | | | | | | | | | |
| Jonsson, 2007^c^ | 70-74 | 18 | 37 | 176,188 | 9,959 | 18 | 39 | 192,120 | | 13,235 | 0.97(0.62,1.52) |
| Van Dijck, 1997 | 68+ | 13 | 51 | 17,487 | 6,502 | 13 | 40 | 16,383 | | 7,261 | 0.80(0.53,1.22) |
| 1. Breast cancer mortality reported is for incidence-based mortality, excluding cases diagnosed before initiation of the population screening program. | | | | | | | | | | | |
| 1. Relative risk calculated from number of breast cancer deaths and person-years. | | | | | | | | | | | |
| 1. Jonsson et al analyzed regions which overlapped with Hellquist paper. Because Jonsson et al provided by age categories (40-49, 50-69, 70-74), we have separated this manuscript into three separate studies. The data for women 40-49 was not included because it overlapped with the Hellquist paper. However, data 50-69 and 70-74 was included and treated as 2 separate studies. | | | | | | | | | | | |
| 1. Person-years provided by corresponding author for original article. | | | | | | | | | | | |
