# Supplementary material for: Screening Mammography & Breast Cancer Mortality: Meta-Analysis of Quasi-Experimental Studies
Source: PLoS One. 2014 Jun 2;9(6):e98105. doi: 10.1371/journal.pone.0098105 (PMC4041743; doi:10.1371/journal.pone.0098105)
Supplement: Table S6 — Data extracted from geographical-historical interaction designs of breast cancer screening programs. (DOC) [file pone.0098105.s008.doc]

| **Table S6. Data extracted from geographical-historical interaction designs of breast cancer screening programs** | | | | | | | | | | | | | | | | | | |
| --- | --- | --- | --- | --- | --- | --- | --- | --- | --- | --- | --- | --- | --- | --- | --- | --- | --- | --- |
| Geographical Comparison |  | | Non-screened Areas | | | | | | | | Screened Areas | | | | | | | |
| Historical Comparison |  | | Historical, non-screened time period | | | | Current, non-screened time period | | | | Historical, non-screened time period | | | Current, screened time period | | | | |
| Author, Year of publication | # of Study Years^a^ | | # Breast Cancer Deaths^b^ | Person-Years | Average Annual Population | | # Breast Cancer Deaths^b^ | Person-Years | Average Annual Population | | # Breast Cancer Deaths^b^ | Person-Years | Average Annual Population | # Breast Cancer Deaths^b^ | | Person-Years | Average Annual Population | |
| **Screened ages <50** | | | | | | | | | | | | | | | | | | |
| Jonsson, 2000^c,d^ | 11 | | 335 | 2,690,000 | 237,279 | | 476 | 3,382,000 | 237,279 | | 183 | 1,783,000 | 202,152 | 235 | | 2,228,000 | | 202,152 |
| **Screened ages 40-64** | | | | | | | | | | | | | | | | | | |
| Jonsson, 2003^c,e^ | 22 | | 4,018 | 12,941,000 | | 618,342 | 3,907 | 12,619,000 | | 618,342 | 280 | 957,000 | 43,749 | 239 | | 885,000 | 43,749 | |
| **Screened ages 50-69** | | | | | | | | | | | | | | | | | | |
| Jonsson, 2001^c,d^ | 11 | | 369 | 1,296,000 | | 98,608 | 318 | 1,264,000 | 98,608 | | 569 | 2,046,000 | 161,986 | 451 | 2,036,000 | | 161,986 | |
| Olsen, 2005^f^ | 10 | | 2,123 | 4,055,004 | | 405,500 | 2,333 | 4,396,417 | 439,642 | | 438 | 634,224 | 63,422 | 223 | 430,823 | | 43,082 | |
| Olsen, 2013^g^ | 13 | | 638 | 1,617,023 | | 124,386 | 523 | 1,576,270 | 121,252 | | 404 | 1,152,755 | 88,673 | 314 | 1,182,747 | | 90,981 | |
| Parvinen, 2006^f,h^ | 15 | | 328 | 618,415 | | 41,228 | 322 | 549,331 | 36,622 | | 94 | 199,329 | 13,289 | 62 | 204,896 | | 13,660 | |
| **Ages Screened 70+** | | | | | | | | | | | | | | | | | | |
| Jonsson, 2003^c^ | | 10 | 141 | 534,000 | | 41,608 | 146 | 580,000 | 41,608 | | 327 | 1,163,000 | 83,830 | 325 | 1,251,000 | | 83,830 | |
| 1. Study years combines the years of accrual and follow-up. Within each study, the follow-up years could have differed. Therefore, we list the average years of study and follow-up reported by the original study. 2. Breast cancer mortality reported is for incidence-based mortality, excluding cases diagnosed before initiation of the population screening program. 3. Unique population numbers were not provided for both the historical and current time period. We used the same population numbers for the both the current and historical periods. 4. Person-years extracted from the original study article slightly differed from what we totaled. We report total person-years that we calculated. 5. This paper tested the effects of screening in Gavleborg, Sweden and had two control regions – neighboring counties to Gavleborg or all of Sweden. For this analysis, we compared Gavleborg to the remainder of the Swedish population. The same number of women in the population was used for both the reference and the study period. 6. Population numbers were not reported in the publication. We calculated the population size by dividing the person-years by number of years of follow-up. 7. We could not obtain the exact RR calculations using data extracted from the paper. The original study authors used Poisson model with 5-year age group, period, region and exposure parameters. As we did not have the original dataset, we re-calculated the relative risk ratios from the data provided in Table 1 of the original study. Original RR was 0.93 (0.77, 1.12). 8. RR and CI reported in this table are only for the comparison of Turku to Helsinki. The comparison of Turku to Tampere was not included because of overlap and because Tampere screened women 55-59. | | | | | | | | | | | | | | | | | | |
